# Abstract of Proceedings of the Buffalo Medical Association

**Published:** 1868-10

**Authors:** Thos. M. Johnson

**Affiliations:** Sec’y.


BUFFALO

### Medical and Surgical Journal.

VOL. VIII. OCTOBER, 1868. No. 3.

### Original Communications.

ART. I.—*Abstract of Proceedings of the Buffalo Medical Association.*

TUESDAY EVENING, August 4th, 1868.

The meeting was called to order by the President. Members present—Drs. J. R. Lothrop, Shaw, Ring, Abbott, Potter, Gay, Wetmore and Johnson.

Dr. Charles B. Schuyler was elected a member on compliance with the by-laws.

DR. POTTER related the following case:

Mr. ——, aged 21, consulted me on the 3d instant, concerning a very annoying discharge from his urethra. The discharge was muco-purulent in appearance and quite abundant, soiling from two to five cloths during the day, according to the severity of his exercise. It was likewise more abundant when his passions were aroused. It is now, and has always been, unaccompanied by pain during micturition and unconnected with erections of the penis. The organ does not now manifest nor has it at any time manifested any signs of acute inflammation. The health and spirits of the patient were much effected by his condition. He assured me that previous to December last he had never enjoyed sexual intercourse, but that he then began a career of excessive venery, which was continued until about the first of April, or during a period of a little more than three months, when the discharge from which he sought relief first made its appearance. Not varying very much in amount or appearance, it has therefore continued four months. Microscopical examination shows absence of either spermatozoa or pus globules, and the presence of certain not well-defined scales, which were thought to be of epithelium. But I have been unable to learn the nature of this discharge, and have regarded it as a result of genital debility, having its origin in excessive venery, and not as a result of a gonorrhœa. Not having met with a similar case before, I mention the history of this man simply to see if my diagnosis is confirmed by the experience of my elder brethren.

Dr. Abbott remarked that he examined with the microscope the substance discharged from the urethra in case just mentioned by Dr. Potter, and was satisfied that it was albuminoid in character.

Dr. Shaw reported the following case:—Was called to see a French woman, about 40 years of age, rather frail, and of nervous temperament. She had been out the day previous and had been over-heated, and on arising in the morning felt unwell, but partook of a moderate breakfast, soon after which she fell upon the floor unconscious. Her family believed she had an attack of paralysis. I found the patient shortly after the attack greatly exhausted, almost pulseless, extremities and surface cold, pupils dilated, could scarcely articulate. Gave stimulants freely; after which she revived, and stated that she at the onset had severe headache, succeeded by dizziness; complained of numbness of the left extremities and side. Applied counter-irritants to the spine; gave tonics and stimulants and am now applying electro-magnetism, under which her condition is improving. I do not regard this as exactly a case of sun-stroke, but think this condition was caused by exhaustion from heat.

Dr. Johnson remarked that he had seen a statement in the newspapers of this city that over forty cases of sun-stroke had occurred here during the present heated term, and thought that this was too large an estimate, that probably several of the cases reported sun-stroke were not genuine cases of that disease. He had seen five persons said to be suffering from sun-stroke. Of these, two cases only were genuine. Of the remaining three cases the first was a case of hysteria; the second an epileptiform convulsion, and the third alcoholic intoxication.

Dr. J. also reported a case of temporary insanity, caused by sun-stroke. The patient was a man of about 35 years of age, whose health had formerly been good. He was a canal-boat captain, and while engaged upon his boat coming through the locks at Lockport, was prostrated by sun-stroke, was properly treated for that disease, and remained insensible for twenty-four hours; arriving in this city at about this time, he left his boat and was found upon the street, and supposed to be suffering from delirium tremens. A man upon the boat with him who had known him and his parents and family for ten years, assured me that the man was strictly temperate, and that there was not now nor had there been any insanity in the family. After being under treatment forty-eight hours he had nearly recovered, and was conveyed home. This was clearly a case of temporary insanity produced by exposure to heat of the sun.

Dr. Ring said that he had seen two cases of sun-stroke, or exhaustion from heat that occurred when the sun was not shining. First case was an old lady on N—— street, just after dark, with the usual symptoms of sun-stroke. Applied cold to the head and gave stimulants, and she recovered. Second case; saw a man next morning, before sunrise, with same symptoms. Applied same treatment, and the man recovered.

Dr. Lothrop thought that direct exposure to the rays of the sun was not essential to the production of that condition called sun-stroke. Excessive heat would produce it, though a great majority of cases arose under direct exposure. In regard to treatment, no one method would suit all cases. In fact, two distinct classes of cases are observed. In one, the symptoms resemble those of apoplexy, while in the other the symptoms are those of exhaustion. The former have the full, slow pulse, the turgid face, the stupor and stertor of apoplexy; the latter, the feeble, often quick pulse, and the pallor of exhaustion. Probably in most instances sun-stroke is nervous exhaustion from excessive heat, and therefore in most cases stimulants with cold to the head would be the best measures. But some cases must be treated upon the antiphlogistic plan, or mischief would be done. The pulse furnishes a good indication of the class to which a case belongs. The distinction is more important in regard to using or withholding stimulants than concerning the application of cold to the head. In all cases the latter is proper, as in all cases there is a marked congestion of the membranes of the brain.

Dr. Lothrop presented two photographic representations of subclavian aneurism with an account of the case. A full report of it could be found in the late work of Dr. John Mason Warren, but as the details of it in that work might not be seen by many of the members, he would briefly relate its main features. A colored man about forty years of age came to his notice, with a tumor in the clavicular region. At that time there were no signs of aneurism except swelling. He subsequently learned that the man had been in the Massachusetts General Hospital, where the diagnosis was subclavian aneurism. The treatment had been applications of ice and pressure of bags of shot. This was employed during the patient's stay, about two months. He then left and about three months after came under Dr. L.'s care. The large tumor was the seat of great pain, so that he could not lie upon the side affected. The pain he attributes to a fall, which happened shortly before, the shoulder being struck. After a time the pain subsided, but the tumor pointed like an abscess, and finally opened and discharged first, chocolate colored pus in large quantity, and afterwards yellow pus. The opening took place over about the middle point of the clavicle, at which point the clavicle was not entire—a separation having been effected either by fracture or absorption. The patient, after about four months was lost sight of; at that time the swelling had entirely subsided, the purulent discharge had nearly ceased, and the man's health had greatly improved. A year afterwards he was heard of, being then in good health. The arm was powerless both before and after the rupture of the tumor and was kept in a sling. After the opening of the tumor, there was considerable depression in the space occupied by it.

This remarkable case was one of cured aneurism, with suppuration subseqently excited on receiving a fall. When first seen by Dr. L. it was an abscess in a closed aneurismal sac. It is difficult to say what was the agency in the cure. Whether coagulation by cold or pressure, either exerted directly upon the sac by the bags of shot, or by the combined pressure of the shot bags and the tumor itself upon the vessel. A tumor may by its bulk alone press upon the vessel and stop the current of blood. External pressure would aid this method of cure. It might have resulted from the closing of the aperture in the vessel by a detached portion of the coagulum in the sac, but this seems less probable than that it was due to cold and pressure acting together.

The suppuration within the sac does not appear to have been connected with the means employed to bring about the cure. We cannot say that it was a result of either pressure or cold, especially as it occurred at a long interval afterwards. It probably resulted from the injury done to the sac by the fall, of which mention has been made. It seems probable, also, that at the same time, the clavicle, weakened by interstitial absorption, the result of pressure, was broken.

The cure of subclavian aneurism, either spontaneously, or by measures other than ligature, is well known to take place. Dr. L. stated that he had now under observation a case of what he thought to be spontaneous cure of aneurism of the left subclavian. In this case there was a tumor in the post clavicular space, but it had neither the thrill, the murmur, nor any of the peculiar signs of an aneurism. For several years there had been but little change in it. The patient, a lady, had been told by eminent surgeons, years before, that it was an aneurism, and had enforced great caution as to her actions. She had lived under a constant apprehension of sudden death. For the last year or two she had been less careful and less apprehensive, but still had considered the tumor to be aneurismal. After a careful examination, Dr. L. found no existing indication of its being an aneurism, and could only conclude that, the diagnosis being assumed to have been correct, it was a case of spontaneous cure. In fact, he thought that the tumor could only be accounted for upon the supposition of its being a cured aneurism.

Dr. Gay made the following remarks upon the influence of the hot weather upon women in the parturient state. Had during the last month attended eleven cases of delivery. Three of these were cases of miscarriage and two cases of delivery by forceps. In one of the cases which occurred when the heat was very oppressive, the first stage of the labor went on naturally, but the second stage was prolonged and tedious. In two primipera cases the os dilated rapidly, but the exhaustion was so great as to retard the labor and make artificial delivery necessary. In one case the exhaustion was so great as to make immediate delivery by forceps necessary. Gave chloroform to complete anæsthesia. The anæsthesia continued until the placenta was expelled, when dangerous hemorrhage came on, accompanied with syncope. I feared the patient would die, and gave stimulants, (brandy,) after which she revived and made a good recovery. Gave first brandy, then ergot, then chloroform.

Dr. Wetmore asked if it was Dr. Gay's practice to give ergot just before introducing the forceps?

Dr. Gay replied, that he had no definite rule. Gave the ergot in this case with the intention of getting its effect as soon as the forceps were introduced, hoping that thereby but little traction would be necessary.

Dr. Lothrop inquired if ergot had been given before the forceps were resorted to? He did not ask the question from any desire to criticise the proceeding of Dr. Gay, even if it had not been given, as many practitioners prefer to use the forceps rather than ergot. It was not a settled matter which was preferable. In a case where everything was ready for the exit of the head, uterine power alone being wanting, there could be no doubt of the propriety of giving ergot, without calling up the question of preference. Its action to increase uterine power could not be called in question. He wished, however, in this connection to speak of a method of giving ergot which had been taught him by a preceptor, which he had followed more or less since. His teacher's theory was, that by producing a firm contraction of the uterus immediately after the expulsion of the placenta, the duration of the lochia was shortened. Accordingly he gave ergot several times, at intervals of an hour, immediately after the birth of the child. Its action was to cause the uterus to become firmly contracted and to prevent the accumulation of coagula in its cavity, whereby not only the lochia were prolonged, but the after pains were also. He kept a record of the duration of the lochia in a large number of cases, and he thought his statistics proved the correctness of his theory. Dr. L. had always thought that the giving of ergot in this way did actually lesson the after-pains and shorten the period of the lochial discharge. Perhaps this in addition might be said in its favor, that it offered a certain amount of protection against excessive flow.

Dr. Ring remarked that he always gave ergot when he feared that hemorrhage might occur, and always gave it with confidence that uterine contraction would be produced from its use.

Dr. Shaw remarked that he had so little faith in the power of ergot that he scarcely ever gave it.

Cholera infantum, cholera morbus, diarrhœa and dysentery were reported as prevailing. Adjourned.

Thos. M. Johnson, Sec'y.

